# Bioinformatics analysis of mRNA and miRNA microarray to identify the key miRNA-mRNA pairs in cisplatin-resistant ovarian cancer

**DOI:** 10.1186/s12885-021-08166-z

**Published:** 2021-04-23

**Authors:** Bai Xue, Shupeng Li, Xianyu Jin, Lifeng Liu

**Affiliations:** 1 Department of Gynecology and Obstetrics, Dalian Municipal Central Hospital, Affiliated to Dalian Medical University, Dalian, 116011 China; 2 Department of Neurosurgery, The Second Affiliated Hospital of Harbin Medical University, Neuroscience Institute, Heilongjiang Academy of Medical Sciences, Harbin, 150086 China

**Keywords:** Cisplatin-resistant, Ovarian cancer, miRNA, mRNA, Bioinformatics analysis, miRNA-mRNA pairs

## Abstract

**Background:**

Ovarian cancer (OC) is a gynecological malignancy with the highest mortality rate. Cisplatin (DDP) based chemotherapy is a standard strategy for ovarian cancer. Despite good response rates for initial chemotherapy, almost 80% of the patients treated with DDP based chemotherapy will experience recurrence due to drug-resistant, which will ultimately result in fatality. The aim of the present study was to examine the pathogenesis and potential molecular markers of cisplatin-resistant OC by studying the differential expression of mRNAs and miRNAs between cisplatin resistant OC cell lines and normal cell lines.

**Methods:**

Two mRNA datasets (GSE58470 and GSE45553) and two miRNA sequence datasets (GSE58469 and GSE148251) were downloaded from the Gene expression omnibus (GEO) database. Differentially expressed genes (DEGs) and differentially expressed miRNAs (DEMs) were screened by the NetworkAnalyst. Gene Ontology (GO) analysis and Kyoto Encyclopedia of Genes and Genomes (KEGG) pathway analysis were conducted to analyze the biological functions of DEGs. The protein-protein interaction network was constructed using STRING and Cytoscape software to identify the molecular mechanisms of key signaling pathways and cellular activities. FunRich and MiRNATip databases were used to identify the target genes of the DEMs.

**Results:**

A total of 380 DEGs, and 5 DEMs were identified. Protein–protein interaction (PPI) network of DEGs containing 379 nodes and 1049 edges was constructed, and 4 key modules and 24 hub genes related to cisplatin-resistant OC were screened. Two hundred ninety-nine target genes of the 5 DEMs were found out. Subsequently, one of these 299 target genes (UBB) belonging to the hub genes of GSE58470 and GSE45553 was identified by MCODE and CytoHubba,which was regulated by one miRNA (mir-454).

**Conclusions:**

One miRNA–mRNA regulatory pairs (mir-454-UBB) was established. Taken together, our study provided evidence concerning the alteration genes involved in cisplatin-resistant OC, which will help to unravel the mechanisms underlying drug resistant.

## Background

Ovarian cancer (OC), originated in gynecological genital tract, is the ninth most common cancer in female with 313,959 newly diagnosed cases and 207,252 new death in 2020 worldwide [1]. OC is more frequently diagnosed at an advanced stage because of the lack of efficient screening measures [2, 3]. And the advanced stages prognosis (FIGO stage III and IV) is extremely poor with 5-year survival rates of approximately 39 and 17%, respectively [4]. The standard treatment of OC involves cytoreductive surgery and platinum-based chemotherapy. Despite the good response rates to initial surgery and chemotherapy, for over a decade, the median progression-free survival rate of patients remains low at about 18 months [5]. Indeed, almost 80% of the patients experience recurrence as a drug resistant population in tumors that ultimately results in fatality [6].

Cisplatin is the first generation of platinum-based drugs that can directly interact with DNA of cancer cells to prevent DNA synthesis and RNA transcription [7]. Numerous protein-coding genes were revealed that are connected with ovarian cancer cisplatin resistance. For example, PTGER3 overexpression confers to cisplatin resistance in OC through up-regulation of Ras-MAPK/Erk-ETS1-ELK1/CFTR1 axis [8]. CpG island promoter hypermethylation of BRCA1, PTK6, PRKCE can enhance sensitivity to cisplatin in OC [9, 10]. Genes with coding ability only account for 2% of all genes in human genome, while the rest belong to noncoding RNAs including microRNA (miRNA), long noncoding RNA (lncRNA), and so on [11]. MicroRNAs (miRNAs) are endogenous, highly conserved small RNAs, 20–24 nucleotides in length, and specifically bind to target mRNA to inhibit post-transcriptional gene expression. MiRNA were also reported to play an important role in cisplatin resistance. MiR-375 overexpression can enhance the cisplatin sensitivity in OC by targeting PAX2 [12]. MiR-138-5p downregulation promotes overexpression of EZH2 and SIRT1 in OC cell, thereby regulating the cisplatin sensitivity [13].

In the present study, bioinformatics tools were used to analyze the cisplatin resistant OC expression profile chips in a public gene chip database for the purpose of identifying differentially expressed genes (DEGs), differentially expressed miRNAs (DEMs), and of constructing miRNA–mRNA regulatory networks involved in cisplatin-resistant OC.

## Materials and methods

### Microarray data

We screened the gene chip data by using the Gene expression omnibus (GEO, http://www.ncbi.nlm.nih.gov/geo) database [14]. Cisplatin resistant ovarian cancer public gene expression data sets (GSE58470 and GSE45553) and miRNA expression data sets (GSE58469 and GSE148251) were selected and downloaded from GEO, with the keywords “ovarian cancer” and “cisplatin resistant” [15–17]. The GSE58470 and GSE45553 datasets, which are two mRNA datasets, contain 2 ovarian cancer cell lines (IGROV-1, OVCAR-8) and 14 samples, including 7 cisplatin resistant cell samples and 7 parental cell samples. The miRNA GSE58469 and GSE148251 datasets, which were analyzed using MicroRNA expression beadchips, contain 2 OC cell lines (IGROV-1, W1) and 12 samples, including 6 cisplatin resistant cell samples and 6 parental cell samples.

### Differentially expressed genes (DEGs) and differentially expressed miRNAs (DEMs)

The raw data of GSE58470, GSE45553 and GSE58469, GSE148251 were effectively processed using the NetworkAnalyst, an online data-analytics platform, using correction, normalization and log2 conversion [18]. The DEGs in cisplatin resistant OC cell lines compared with parental cell lines were determined using limma algorithm [19]. DEGs were screened with a false discovery rate (FDR) corrected *p*-value< 0.05 and |log2 fold-change (FC)| > 1. The DEMs in cisplatin resistant OC cell lines compared with parental cell lines were also processed by the NetworkAnalyst. The FDR corrected *p*-value< 0.05 and |log2 fold-change (FC)| > 1 were used as the screening thresholds.

### Functional enrichment analysis of DEGs

DAVID (https://david.ncifcrf.gov/), a widely used web-based genomic functional annotation tool, was used for data annotation analysis [20]. In our study, we used the DAVID to perform Gene Ontology (GO) analysis including cellular component (CC), molecular function (MF), biological process (BP), and Kyoto Encyclopedia of Genes and Genomes (KEGG) pathway enrichment analysis. A *p*-value that is smaller than 0.05 and enrichment score above 1.5 were considered as significant enrichment.

### Construction of protein-protein interaction (PPI) networks and module research

The PPI network of the DEGs was constructed and visualized using the STRING (https://string-db.org) database to determine the molecular mechanisms of key signaling pathways and cellular activities in cisplatin resistant ovarian cancer [21]. An interaction score > 0.4 was considered to identify the significant PPIs. We then used the Cytoscape software (version 3.8.2; www. cytoscape.org) to analyze the PPI network [22]. Relationships among DEGs were analyzed by NetworkAnalyzer plug-in of Cytoscape software to characterize small-world network through calculating the network properties such as the clustering coefficient of the network, distribution of node degree and the shortest path [23]. Molecular Complex Detection (MCODE) was used to identify key clusters of genes within PPI network using the cutoff criteria (MCODE score > 5) [24, 25] with the default parameters (degree cutoff = 5, node score cutoff = 0.2, K-core = 2, and Max depth = 100). Hub genes in the network were selected using CytoHubba through connection degree method (cutoff criteria: degree above 20). Finally, we summarized the overlapping genes between results of MCODE and CytoHubba to create a consensus of predictions to identify more accurate hub genes.

### miRNA target prediction

MiRNATip and FunRich are two bioinformatics platform and analysis tools for predicting DEMs target genes and miRNA-gene pairs [26–28]. In the present study, the targets of the DEMs were predicted using MiRNATip and FunRich (version 3.1.3). The Venny 2.1 online Tool (http://bioinfogp.cnb. csic.es) was used to find overlapping genes between DEGs and predictive targeted genes of DEMs [29]. The miRNA-gene regulatory network was depicted and visualized using Cytoscape software.

### Construction of miRNA–mRNA regulatory pairs

MiRNA–mRNA regulatory pairs related to cisplatin-resistance in ovarian cancer were constructed according to the miRNA targets prediction results and DEGs to display the interaction among miRNA and mRNA. Furthermore, the K-M plotter database (https://kmplot.com/analysis/) and OncomiR database (http://www.oncomir.org/) were used to assess the potential prognostic significance of our selected mRNAs and miRNAs by performing survival analysis [30, 31].

## Results

### Identification of DEGs, DEMs

**Fig. 1:**
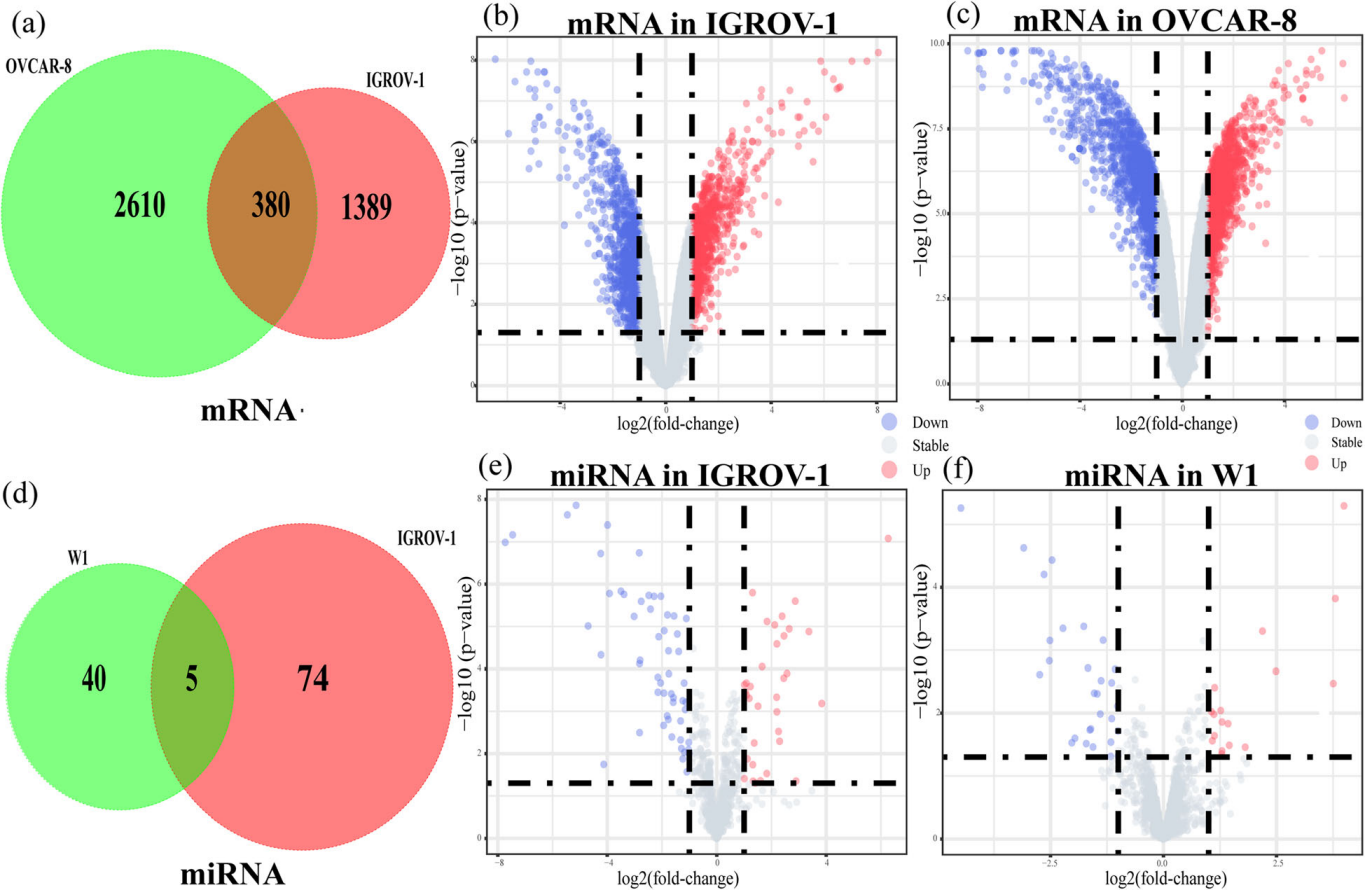
Identification of overlapping DEGs and DEMs related to cisplatin-resistant OC. **a** Venn diagram for the overlapping DEGs between OVCAR-8 and IGROV-1. **b** Volcano plots for DEGs in IGROV-1. **c** Volcano plots for DEGs in OVCAR-8. **d** Venn diagram for the overlapping DEMs between W1 and IGROV-1 cell lines. **e** Volcano plots for DEMs in IGROV-1. **f** Volcano plots for DEMs in W1

The data was successfully normalized to ensure the accuracy. Dataset GSE58470 and GSE45553 were utilized to identify DEGs in IGROV-1 and OVCAR-8 cell lines. As presented in Fig. 1a, a total of 380 overlapping DEGs were identified between IGROV-1 and OVCAR-8 cell lines. Volcano plots were drawn to give a direct presentation of all the examined genes in the data sets. The red dots represent the significantly upregulated genes, while the blue dots represent the significantly downregulated genes. Volcano plots for gene expression in IGROV-1 and OVCAR-8 cell lines were presented in Fig. 1b and c. In miRNA datasets GES58469 and GSE148251,5 overlapping DEMs were detected between IGROV-1 and W1 cell lines which was shown in Fig. 1d. Volcano plots for miRNA expression in IGROV-1 and W1 cell lines are presented in Fig. 1e and f.

### Functional enrichment analysis of DEGs

**Fig. 2:**
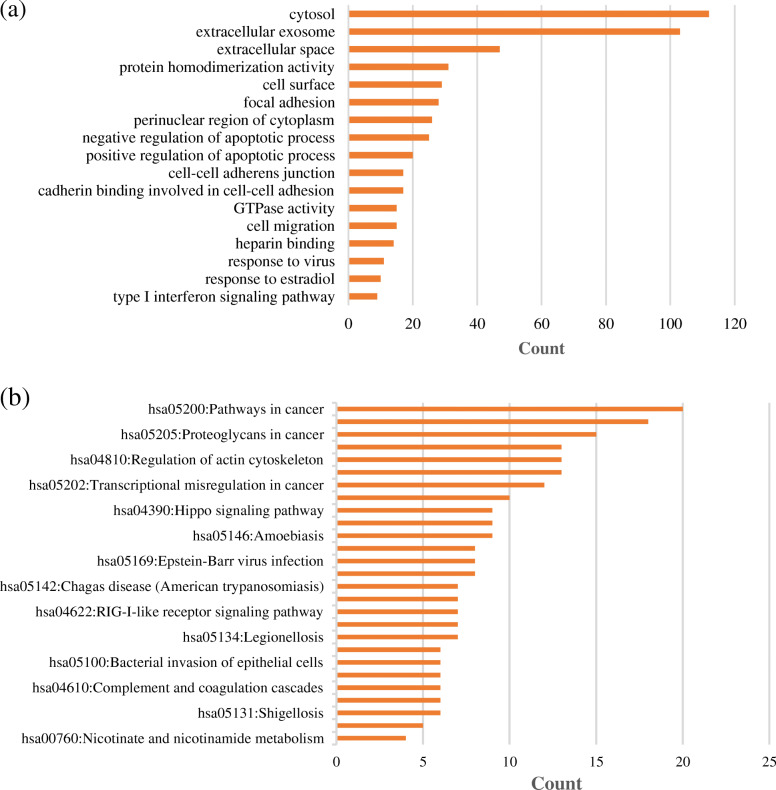
Functional enrichment analysis of DEGs. **a** GO analysis of DEGs in GSE58470 and GSE45553 datasets. **b** KEGG analysis of DEGs in GSE58470 and GSE45553 datasets

GO analysis including cellular components (CC), molecular function (MF), biological process (BF), and KEGG analysis was performed using DAVID database to understand the functions of DEGs. The GO functional enrichment analysis resulted in a total of 380 DEGs mapped to 265 GO terms. With the FDR corrected *p*-value< 0.05 and enrichment score > 1.5 as the cut-off value, 17 significant enriched functional clusters were screened (Fig. 2a). In total, 7 GO terms were significantly enriched in cellular components including ‘extracellular exosome’, ‘cytosol’, ‘cell surface’, ‘extracellular space’, ‘cell-cell adherens junction’, ‘focal adhesion’ and ‘perinuclear region of cytoplasm’. Enrichment of 4 GO terms, such as ‘heparin binding’, ‘GTPase activity’, ‘cadherin binding involved in cell-cell adhesion’ and ‘protein homodimerization activity’, belongs to molecular functions. A total of 6 biological processes were enriched, mainly involving ‘type I interferon signaling pathway’, ‘response to estradiol’, ‘response to virus’, ‘cell migration’, ‘positive regulation of apoptotic process’ and ‘negative regulation of apoptotic process’. A total of 380 DEGs were mapped into the KEGG database using DAVID, enrichment score > 1.5 and *p*-value< 0.05 were used as an enrichment screening standard. In total, 27 enriched functional clusters of the DEGs were obtained (Fig. 2b), such as ‘Pathways in cancer’ (20 genes), ‘PI3K-Akt signaling pathway’ (18 genes), ‘Proteoglycans in cancer’ (15 genes), ‘MAPK signaling pathway’ 13 genes), ‘Regulation of actin cytoskeleton’ (13 genes).

### Establishment of PPI network and identification of hub gene

**Fig. 3:**
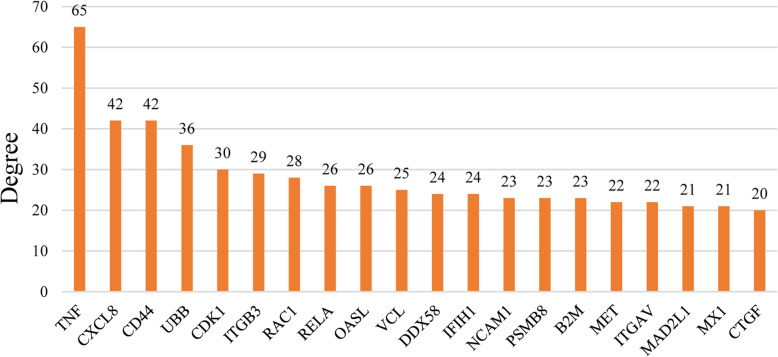
Degree of Top 20 genes in cisplatin resistant OC

**Fig. 4:**
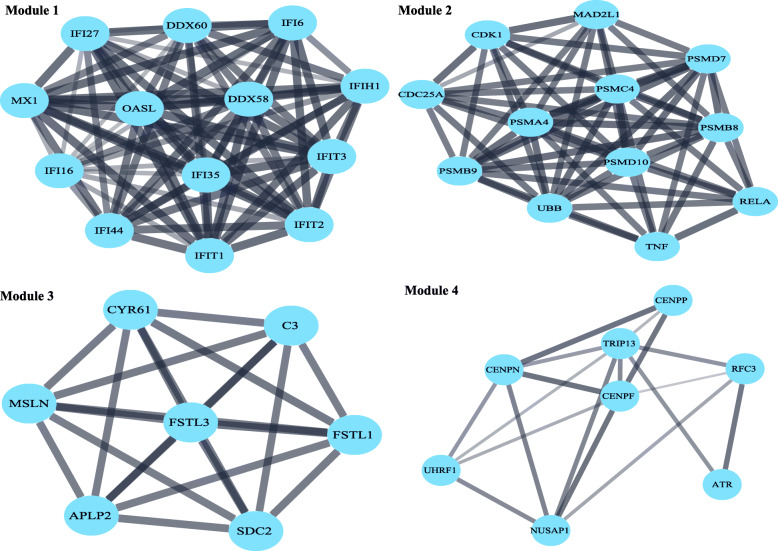
Four key modules of the protein-protein interaction network analysis

**Fig. 5:**
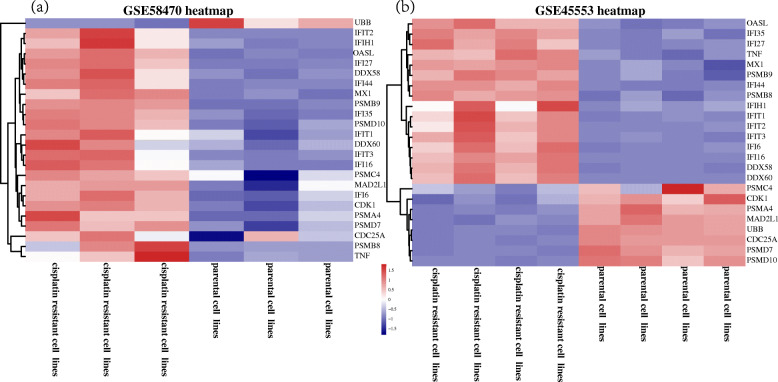
heatmap of the 24 hub genes. **a** 24 hub genes of GSE58470. **b** 24 hub genes of GSE455553

The PPI network of 380 DEGs was constructed and visualized using STRING database. The isolated nodes and partially loosely connected gene nodes were removed, and the remaining DEGs together constituted a complex multi-center interaction network map, which contained 379 nodes and 1049 edges. The average node degree was 5.54 and the average local clustering coefficient was 0.394. Among the 379 nodes, top 20 DEGs with the highest degree of nodes were screened based on the Cytoscape software analysis results (Fig. 3). The results of the top 10 DEGs were as follows: TNF, CXCL8, CD44, UBB, CDK1, ITGB3, RAC1, RELA, OASL, VCL. The key clusters of genes were obtained using MCODE, with 9 key modules and a false degree cutoff = 5. Four significant key modules including 40 key genes with the MCODE score > 5 were identified (Fig. 4). Subsequently, the CytoHubba was used to find the hub genes in the PPI network of the DEGs. In total, 25 hub genes were identified. At last, we summarized the overlapping genes between the MCODE and CytoHubba results (Table 1). Twenty-four hub genes belonging to the GSE58470 and GSE45553 were identified. Furthermore, the functional and pathway enrichment of these genes was also analyzed using DAVID online tools shown in Tables 2 and 3. The 24 significantly upregulated or downregulated hub genes were also presented in heatmaps (Fig. 5a,b).

**Table 1:** 24 hub genes of GSE58470 and GSE45553

Gene symbol	Degree	MCC Score
MX1	22	479000000
IFIT1	17	479000000
IFIT2	16	479000000
OASL	28	479000000
IFIT3	15	479000000
DDX58	25	479000000
IFIH1	24	479000000
DDX60	15	479000000
IFI44	13	479000000
IFI35	15	479000000
IFI6	13	479000000
IFI27	13	479000000
IFI16	14	479000000
PSMB8	25	403736
UBB	40	403798
PSMB9	21	403736
PSMC4	19	403475
PSMA4	19	403473
PSMD7	14	403392
PSMD10	14	403332
CDK1	34	364936
MAD2L1	25	364750
CDC25A	22	364044
TNF	68	43334

**Table 2:** GO analysis of 24 hub genes

Category	ID	Description	Count	***P***-value
Molecular function	GO:0005515	Protein binding	21	< 0.001
	GO:0005524	ATP binding	6	0.039
	GO:0003725	Double-stranded RNA binding	4	< 0.001
	GO:0004298	Threonine-type endopeptidase activity	3	< 0.001
	GO:0003727	Single-stranded RNA binding	3	0.001
Cellular component	GO:0005829	Cytosol	19	< 0.001
	GO:0005737	Cytoplasm	17	< 0.001
	GO:0005634	Nucleus	14	0.007
	GO:0005654	Nucleoplasm	10	0.005
	GO:0000502	Proteasome complex	6	< 0.001
Biological process	GO:0009615	Response to virus	10	< 0.001
	GO:0060337	Type I interferon signaling pathway	9	< 0.001
	GO:0051436	Negative regulation of ubiquitin-protein ligase activity involved in mitotic cell cycle	9	< 0.001
	GO:0051437	Positive regulation of ubiquitin-protein ligase activity involved in regulation of mitotic cell cycle transition	9	< 0.001
	GO:0031145	Anaphase-promoting complex-dependent catabolic process	9	< 0.001

**Table 3:** KEGG analysis of the 24 hub genes

Term	Description	Count	***P***-value
hsa03050	Proteasome	5	< 0.001
hsa05168	Herpes simplex infection	5	< 0.001
hsa05164	Influenza A	4	0.004
hsa04622	RIG-I-like receptor signaling pathway	3	0.007
hsa04914	Progesterone-mediated oocyte maturation	3	0.011
hsa05169	Epstein-Barr virus infection	3	0.021
hsa04110	Cell cycle	3	0.022
hsa05162	Measles	3	0.025
hsa05160	Hepatitis C	3	0.025
hsa05161	Hepatitis B	3	0.029

### miRNA-gene regulatory network

**Fig. 6:**
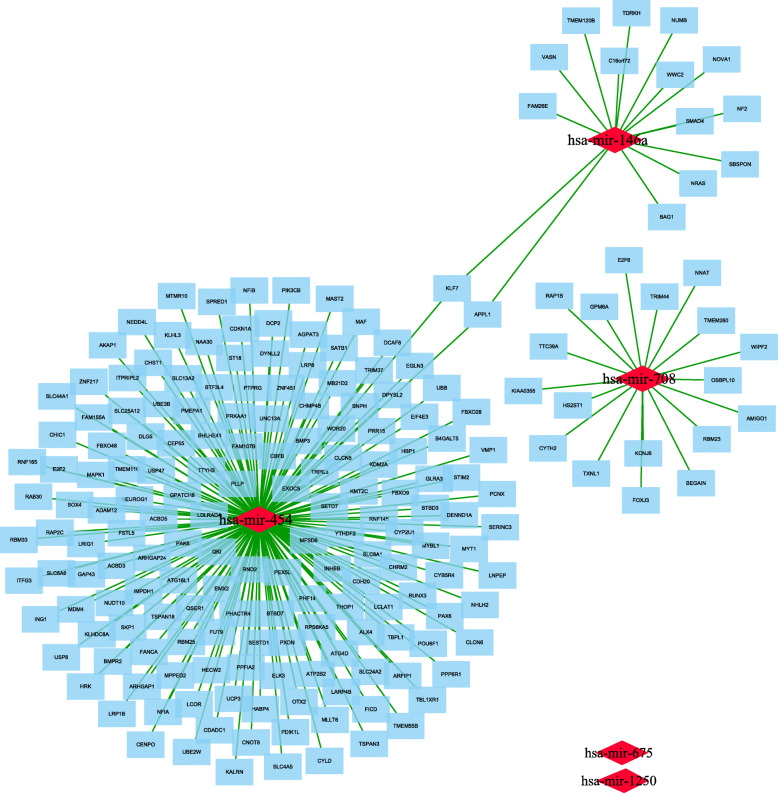
PPI network of 299 target genes, which were regulated by 3 miRNAs

**Fig. 7:**
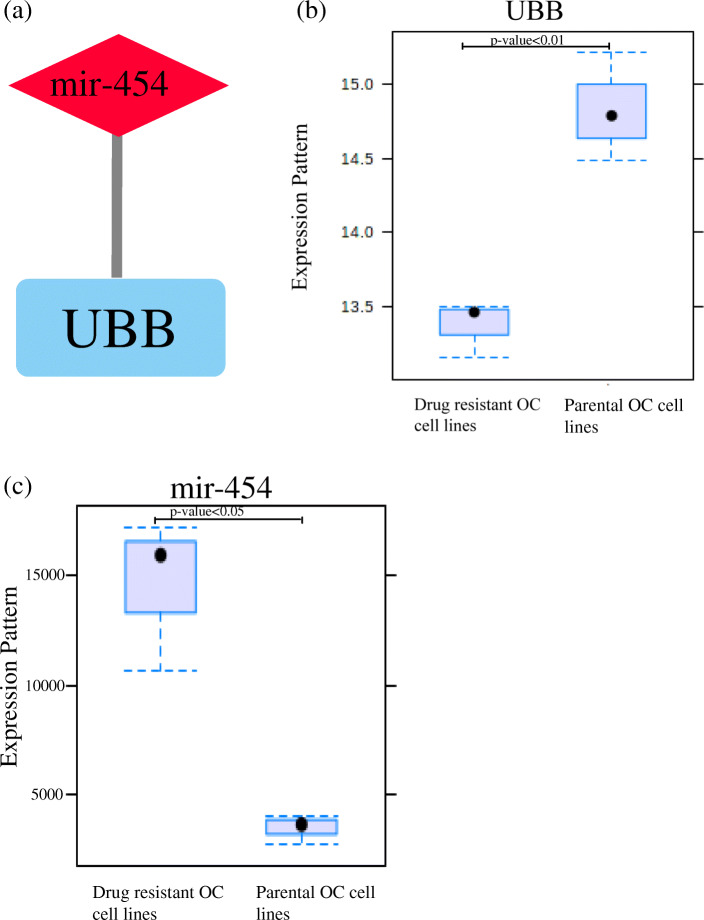
**a** hub gene mRNA UBB was regulated by the miRNA mir-454. **b** the expression of the UBB was significantly different between drug resistant OC and parental OC cell lines. **c** the expression of the mi-454 was significantly different between drug resistant OC and parental OC cell lines

**Fig. 8:**
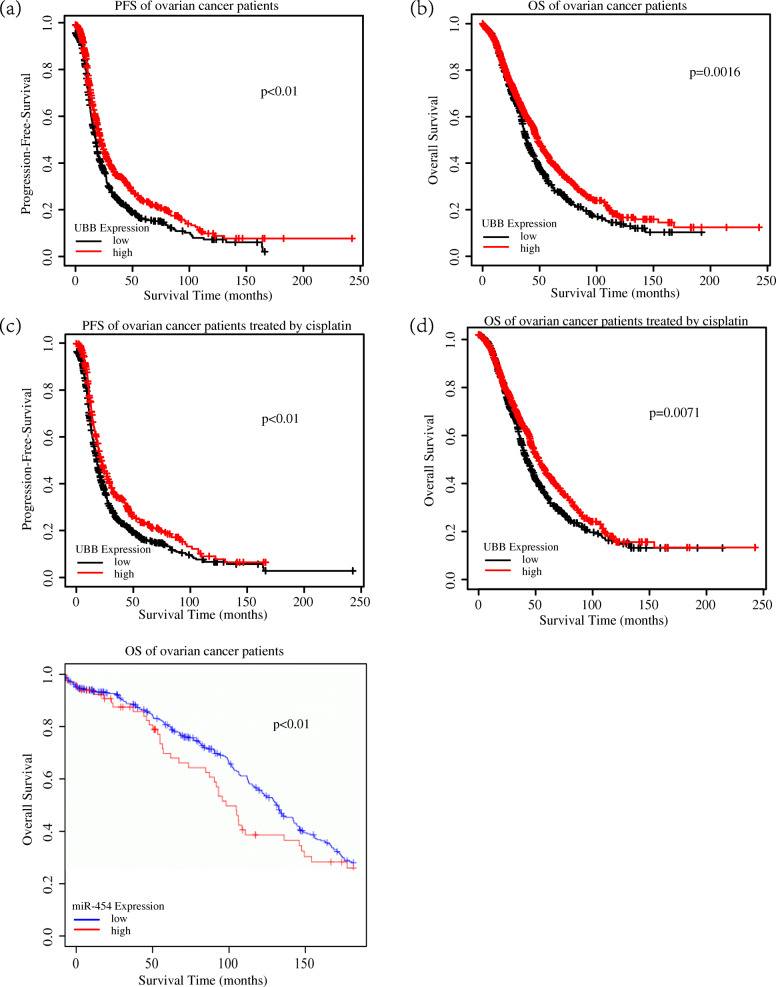
UBB and mir-454 expression was associated with poor prognosis in ovarian cancer. **a**, **b**, **e** Kaplan-Meier survival analysis of ovarian cancer patients. **c**, **d** Kaplan-Meier survival analysis of ovarian cancer patients treated by cisplatin

The FunRich (http://www.funrich.org) online tool searched and predicted 611 target genes of the 5 DEMs and the MiRNATip predicted 1534 target genes of 5 DEMs. And then, there were 299 overlapping genes between the predicted targeted genes of FunRich and MiRNATip. As presented in Fig. 6, the 299 overlapping genes were regulated by 3 DEMs (mir-146a, mir-708, mir-454), but 2 DEMs (mir-675, mir-1250) have no targeted genes. In addition, the target gene UBB among the 299 overlapping genes also belonged to the 24 hub genes of GSE58470 and GSE45553 and was regulated by mir-454 (Fig. 7a). The expression of the UBB was low and the expression of the mir-454 was high in cisplatin resistant cell lines (Fig. 7b,c). Furthermore, to validate the predicting results of our present study, we have performed the survival analysis by using K-M plotter database and OncomiR database. The results of Kaplan-Meier survival analysis illustrated that lower UBB expression predicted poor progression-free-survival and overall survival both in ovarian cancers (Fig. 8a,b) and cisplatin treated ovarian cancers (Fig. 8c,d), respectively. The Kaplan-Meier survival analysis of mir-454 revealed that high expression of the mir-454 predicted poor prognosis in ovarian cancer (Fig. 8e). On the basis of these results, the miRNA–mRNA regulatory pair was established, indicating the importance of this miRNA-mRNA pair in cisplatin-resistant ovarian cancer.

## Discussion

Ovarian cancer is a lethal malignant cancer with different histopathological and biological characteristics, and a < 40% overall remission rate at all stages [32]. Over the past a few decades, systematic chemotherapy has brought plenty of benefits to patients with OC [33]. Cisplatin-based chemotherapy is the most commonly used treatment regimen; however, acquisition of cisplatin resistance is a major clinical obstacle to treatment of OC [34]. Thence, there is a compulsive need to identify more effective biomarkers to overcome cisplatin resistance and improve the prognosis of OC. Noncoding RNAs are reported to have vital roles in regulating human cell behaviors [35, 36]. MicroRNAs (miRNA) are a kind of noncoding RNAs, which are composed of 22–25 nucleotides and bind with the 3′-untranslated region of targeted mRNAs to regulate mRNA expression [37]. miRNAs have been regarded as essential regulators of chemoresistance of many cancers to common chemotherapy agents [38, 39], including cisplatin-based chemotherapy [40, 41].

In our study, 380 DEGs were screened from the GSE58470 and GSE45553 datasets and processed using bioinformatics methods. The results of the KEGG and GO enrichment analysis of the DEGs revealed that the identified genes were enriched in various signaling pathways, such as ‘Pathway in cancer’, ‘Proteoglycans in cancer’, ‘RIG-I-like receptor signaling pathway’, ‘Hippo signaling pathway’. RIG-I-like receptors, a member of cytosolic pattern-recognition receptors (PRRs), can distinguish pathogen-associated molecular patterns and activate antiviral immune responses [42]. The RIG-I-like receptor signaling pathway induce the expression of large numbers of IFN-β and IFN-α proteins [43]. A recent research has illustrated that the downregulation of RIG-I expression was correlated with poorer prognosis and weakened response to IFNα therapy in hepatocellular carcinoma (HCC) patients [44]. The KEGG analysis results of our study revealed that the aberrant activation of the RIG-I like receptor signaling pathway may be related with the cisplatin resistance in the OC patients, which needed to be further validated. Hippo signaling pathway is one of the eight major signaling pathways commonly altered in human cancers [45]. Dysfunction of Hippo signaling pathway has been implicated in an increasing number of human cancers [46]. For example, miR372 and miR373 mediated silencing of LATS2 expression, a Hippo pathway tumor suppressor, is related with testicular germ cell tumors [47]. MiR-149-5p can aggravate chemoresistance in ovarian cancer cells by directly targets the core kinase components of the Hippo signaling pathway [48].

In the present study, 5 overlapping DEMs (mir-146a, mir-708, mir-454, mir-675, mir-1250) were identified from the GSE58469 and GSE148251 datasets. Subsequently, we predicted the target genes of these 5 DEMs by using the FunRich and MiRNATip analysis tools and got 299 different target genes regulated by 3 DEMs (mir-146a, mir-708, mir-454). Among these 299 target genes, UBB also belongs to the 24 hub genes of GSE58470 and GSE45553 which is regulated by mir-454. Stefanie et al. has demonstrated that mir-454 can regulate UBB expression in kidney tissue and HEK293 cell lines [49]. But, the role of mir-454-UBB regulatory pair has not been revealed in cisplatin treated ovarian cancer before. So, we perform our bioinformatics analysis and the results of our present study indicate that the mir-454-UBB regulatory pair is associated with cisplatin resistance in OC. To validate the results of our study, the survival analysis was performed and illustrated that UBB and mir-454 expressions are associated with the prognosis of ovarian cancer. The K-M survival plots proven that low expression of UBB and high expression of mir-454 may not only predict poor prognosis of OC (Fig. 8a,b,e) but also poor prognosis of cisplatin treated OC (Fig. 8c,d), which indicated that mir-454 and UBB regulatory pair may be correlated with cisplatin resistance in OC.

MiR-454 has been reported to be implicated in the progression of many types of cancer and play important roles in chemotherapeutic drug resistance. Several studies show that miR-454 functions as an oncogene in colorectal cancer [50], hepatocellular carcinoma [51], non-small cell lung cancer [52] and induce the oxaliplatin resistance in gastric carcinoma cells by targeting CYLD [53]. UBB also known as ubiquitin B is highly expressed in all eukaryotic cells and can mark some target proteins for ubiquitin-proteasome system degradation. Previous studies emphasize UBB and UBB dependent ubiquitin-proteasomal protein degradation are essential in histone deacetylase inhibitor-induced tumor selectivity. Tian et al. studied the role of UBB expression in cervical cancer and demonstrated that UBB can maintain cancer stem-like characteristics [54]. The results of our current study showed that miR-454 -UBB regulatory pair was significant in cisplatin resistance OC cell lines. This study may advance the understanding of the mechanism of cisplatin resistance in ovarian cancer and suggest that miR-454 and UBB may be two novel biomarker and therapeutic targets for ovarian cancer patients.

There are some limitations in our study. First, the regulatory network we constructed using bioinformatic methods was not validated by experimental work. In our future work, we will validate these findings using experiment manners on cell lines and human tissues. Second, all the data analyzed in our study was retrieved from one online database, which might result in some biases. So, further studies consist of larger sample sizes are needed to validate our findings.

## Conclusions

Our study analyzed gene and miRNA expression between cisplatin resistant OC cell lines and parental OC cell lines using mRNA data and non-coding RNA data from the GEO database, and identified aberrant expression of mRNAs and miRNAs in cisplatin resistant OC cell lines. Based on these analysis results, we found that mir-454-UBB regulatory pair is significant in cisplatin resistance ovarian cancer cell lines. These finds will no doubt help us to understand the mechanisms under the skin of ovarian cancer cisplatin-resistance.

## Data Availability

All data generated or analyzed during this study are included in Gene expression omnibus GEO) public database. The mRNA datasets (GSE58470, https://www.ncbi.nlm.nih.gov/geo/query/acc.cgi?acc=GSE58470 and GSE45553, https://www.ncbi.nlm.nih.gov/geo/query/acc.cgi?acc=GSE45553). The miRNA datasets (GSE58469, https://www.ncbi.nlm.nih.gov/geo/query/acc.cgi?acc=GSE58469 and GSE148251, https://www.ncbi.nlm.nih.gov/geo/query/acc.cgi?acc=GSE148251).

## Abbreviations

OC: Ovarian cancer
DDP: Cisplatin
GEO: Gene expression omnibus
DEGs: Differentially expressed genes
DEMs: Differentially expressed miRNAs
GO: Gene Ontology
KEGG: Kyoto Encyclopedia of Genes and Genomes
PPI: Protein–protein interaction
